# Effects of nutritional supplements in healing of laparotomic dehiscences in obese patients with metabolic syndrome: a randomized prospective controlled study

**DOI:** 10.1186/1471-2318-11-S1-A19

**Published:** 2011-08-24

**Authors:** M Galeotalanza, S Spiezia, M Santangelo

**Affiliations:** 1 Department of Neuroscience, Physiology Nutrition Unit, University Federico II, Naples, Italy; 2 General, Thoracic and Vascular Surgery Department, O.U. of General Surgery and Organ Transplantation, University of Naples "Federico II," Naples, Italy

## Background

Chronic venous insufficiency, infection, diabetes mellitus, excess malnutrition, exposure to pressure and shear, all conditions included in obesity, prolong the healing process. Moreover, a growing body of evidence clearly indicates that dietary supplementation or intravenous administration of Arg is beneficial in facilitating wound healing, enhancing insulin sensitivity, and maintaining tissue integrity. Arginine produces physiologic effects via nitric oxide dependent and independent pathways. Nitric oxide is important for the modulation of vascular tone, inflammation, immune function, endothelial function, platelet and leukocyte adherence, and neurotransmission. Nitric oxide modulates many biochemical processes important for the response to sepsis. Arginine, independent of nitric oxide, is important for growth, wound healing, cardiovascular function, immune function, inflammatory responses, energy metabolism, urea cycle function, and other metabolic processes. Arginine supplementation improves outcomes in animals with sepsis, wounds, ischemia-reperfusion injury, and following thermal injury. Enteral administration of arginine improves endothelial function but has little effect upon hemodynamics during human sepsis. An analysis of clinical studies using enteral formulas with supplemental arginine suggests benefits upon outcome, with no evidence of significant detrimental effects. The aim of this study was to evaluate the healing effects of a normocaloric diet enriched in arginine, eicosapentanoic acid (EPA) and gamma-linolenic acid (GLA) and vitamins (vitamins A, C and E) on laparotomies wound dehiscence.

## Materials and methods

Thirty obese (BMI > 35 kg/m^2^) patients with acute wound infections were included in a study evaluating the effects of protein, lipids and vitamins on healing of wound dehiscence. A diet enriched with nutritional supplements of arginine, lipids (EPA, GLA) and vitamins (vitamins A, C and E) was compared with a diet not enriched. The healing of wound dehiscence was evaluated weekly. Nutritional assessment included levels of serum albumin, C-reactive protein.

## Results

Patient's age, severity of disease and gender distribution were similar in the two groups. The study group had a higher body mass index. At baseline, the wound dehiscences were similar in the two groups. A significant reduction of healing time of existing wounds was observed in the study group compared to the control group (p<0.05). There was no significant difference in the nutritional parameters between the two groups.

## Conclusions

A diet enriched with arginine, EPA, GLA and vitamins A, C and E is associated with a significantly lower healing time of wound dehiscences in critically obese patients
